# Rejuvenation by inhibiting TGF-β1/pSmad signaling

**DOI:** 10.18632/oncotarget.4563

**Published:** 2015-06-22

**Authors:** Elizabeth H. Corder, Karta Purkh Singh Khalsa

**Affiliations:** Complex Biological Systems Alliance, North Andover, MA, USA

Yousef *et al*. [1] report the rejuvenation of myogenesis and neurogenesis in old mice ―24 months old, analogous to 80 year old humans ― by reversing TGF-β1/pSmad signaling to the lower levels found in young mice. They suggest that the higher levels inhibit the capacity of stem cells in old muscle and in the hippocampus of the aged brain to contribute to differentiated tissue. Reversal was accomplished pharmacologically by a single small molecule. These findings may constitute a milestone in aging research.

At this point in time it may be worth taking a moment to step back and acknowledge that traditional medical systems―Westerm, Ayurvedic (Indian) & Chinese―include materica medica, namely plants, whose leaves or roots have been found to reverse TGF-β1/pSmad signaling in experimental systems and patient populations (URL: http://www.ncbi.nlm.nih.gov/pubmed). Here are five examples:

Gotu kola leaf (*Centella asiatica*) used in Ayurvedic and Chinese medicine to promote the rapid healing of wounds without scarring, mitigate fibrotic conditions such as liver cirrhosis and keloids, and to treat neurologic conditions including senility. Recent biomedical reports in a number of experimental systems indicate that Asiatic acid and other constituents block TGF-beta/Smad signaling: liver fibrosis (rat, CCL(4)) [2], cardiac myopathy (mice, pressure overload), pulmonary fibrosis (mice, bleomycin-induced), keloids (cell culture), renal tubulointerstitial fibrosis (mice, obstruction of one ureter), burn wound healing (mice), and atrial fibrillation (AF patients). One plant used almost interchangeably, or in combination, with gotu kola named brahmi (Bacopa monnieri, often described as *B. monniera*) has wide usage in India presently and since ancient times to maintain cognition in older persons, as well as to treat other conditions such as irritable bowel syndrome.

Astralagus root is the premier herb in traditional Chinese medicine to boost the immune system, for example, to ward off winter colds and flu. Astragalus propinquus is the preferred name for Chinese medicinal Astragalus root (syn. Astragalus membranaceus). Like gotu kola, recent experimental reports demonstrate attenuation of TGF-beta/Smad signaling and reduced fibrosis in model systems that investigate astralagus [3, 4].

Dan shen root (*Salvia miltiorrhiza*, “Red sage”), is a mainstay of Chinese medicine to maintain the health of the circulatory system, able to reduce blood sugar levels and prevent the formation of advanced glycation products associated with an elevated risk of cognitive decline, and as a modern Chinese treatment for acute pancreatitis, among other uses. Again, dan shen attenuates TGF-beta/Smad signaling and reduces fibrosis in model systems [3–5].

Turmeric rhizome (*Curcuma longa*) has wide application in both Ayurvedic and Chinese systems and is well known to us as a spice and as the agent that gives mustard its yellow color. The curcumin in turmeric may be the strongest known inhibitor of TGF-β1/pSmad signaling, found in one experimental system to reduce levels in diabetic nephropathy, thereby lessening proteinurea and improving kidney function in a patient sample [6].

Sarsaparilla root (*Smilax glabra*) has long been used to strengthen bones and muscles and to treat infection, among other uses including as a flavoring agent renowned for its pleasant taste. Root extracts inhibit TGF-β1/pSmad signaling, thereby inhibiting the migration of cancer cells in one experimental system [7].

This is not a complete list of plants each having characteristically manifold medicinal uses in traditional medicine able to reverse TGF-β1/pSmad signaling in experimental systems and patient populations. Their long usage suggests relative safety and efficacy, and acceptability at effective doses, in general human populations. They are readily available. Taking this information into account, one implication of Yousef *et al.* [1] is that these widely available food-like herbs might be included in the diet of adults to modulate TGF-β1/pSmad signaling. Moreover, these and other materica medica from traditional medical systems might be investigated as modern therapies to improve stem cell function.
